# Exhaustive extraction of cyclopeptides from *Amanita phalloides*: Guidelines for working with complex mixtures of secondary metabolites

**DOI:** 10.1002/ece3.6191

**Published:** 2020-03-24

**Authors:** Clare H. Scott Chialvo, Logan H. Griffin, Laura K. Reed, Lukasz Ciesla

**Affiliations:** ^1^ Department of Biological Sciences University of Alabama Tuscaloosa AL USA; ^2^ Department of Biology Appalachian State University Boone NC USA

**Keywords:** accelerated solvent extraction, *Amanita phalloides*, cyclopeptides, extraction, HPLC‐MS, plant‐insect interactions, secondary metabolites

## Abstract

1. Understanding plant‐insect interactions is an active area of research in both ecology and evolution. Much attention has been focused on the impact of secondary metabolites in the host plant or fungi on these interactions. Plants and fungi contain a variety of biologically active compounds, and the secondary metabolite profile can vary significantly between individual samples. However, many experiments characterize the biological effects of only a single secondary metabolite or a subset of these compounds.
2. Here, we develop an exhaustive extraction protocol using an accelerated solvent extraction protocol to recover the complete suite of cyclopeptides and other secondary metabolites found in *Amanita phalloides* (death cap mushrooms) and compare its efficacy to the “Classic” extraction method used in earlier works.
3. We demonstrate that our extraction protocol recovers the full suite of cyclopeptides and other secondary metabolites in *A. phalloides* unlike the “Classic” method that favors polar cyclopeptides.
4. Based on these findings, we provide recommendations for how to optimize protocols to ensure exhaustive extracts and also the best practices when using natural extracts in ecological experiments.

## Graphical abstract

While host plants and fungi contain a complex suite of metabolites, many studies examining plant‐insect interactions focus on the biological effect of a single compound. We developed a new protocol to exhaustively extract secondary metabolites from *Amanita phalloides* mushrooms. Based on our findings, we present recommendations of best practices for studies using secondary metabolite extracts.

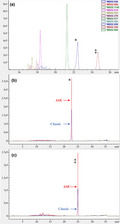

## 1 INTRODUCTION

Understanding plant‐insect interactions has been and remains an active area of chemical ecology (see Dyer et al., 2018; Erb & Reymond, 2019; Mithöfer, Boland, & Maffei, 2018; for recent reviews). The seminal work of Fraenkel (1959) demonstrated that secondary metabolites played an important role in host plant defense, and this finding generated several new lines of inquiry (e.g., How do insects exploit their hosts defense? and What allows some insects to feed on hosts containing highly toxic compounds?). One of the greatest challenges associated with addressing these questions is the diversity of secondary metabolites found in host plants and/or fungi (Ciesla & Moaddel, 2016; Wink, 2008). Unsurprisingly, most studies investigating the effects of secondary metabolites on insects examined the impact of one metabolite (Jones & Agrawal, 2016; Liu, Vrieling, & Klinkhamer, 2017). However, it is important to note that the potent bioactivity of some compounds in their natural matrix is due to synergistic and/or antagonistic interactions between secondary metabolites that are absent when the compound is tested in isolation (Dyer et al., 2003; Richards et al., 2016). In addition, plant‐insect interaction studies that do use secondary metabolite extracts are complicated by the fact that the secondary metabolite profile of extracts varies from sample to sample, depends on many factors (e.g., collection site, drying conditions, and storage) and contributes greatly to the experimental outcome (Ciesla & Moaddel, 2016). Therefore, the observed biological effect from such assays can only be attributed to the specific sample used in the experiment. In addition, correct interpretation of experimental data is also jeopardized by the lack of detailed information related to extraction and secondary metabolite profile of the natural sample (Ladhari, Laarif, Omezzine, & Haouala, 2013; Ventrella et al., 2016; Wachira et al., 2014). Thus, an absence of guidelines when using secondary metabolite extracts in ecological experiments results in a lack of reproducibility of experiments.

In an ideal experimental setup, the organism of interest would be reared in its natural habitat with unlimited access to the host plant or fungus. Additionally, the secondary metabolite profile of each host used in the study would be strictly monitored to correctly interpret the experimental outcomes. However, the implementation of this model is far from realistic.

To address some of the complexity associated with host secondary metabolite extracts and to develop protocols to improve experimental replicability, we examined these questions with regards to obtaining complete extracts of *Amanita phalloides* (death cap mushrooms). These mushrooms contain a mixture of cyclopeptide toxins that are the source of their toxicity (Wieland, 1968a). Three cyclopeptide subclasses (i.e., amatoxins, phallotoxins, and virotoxins) have been identified and differ based on chemical structure (bicyclic octapeptides, bicyclic heptapeptides, and monocyclic heptapeptides, respectively) (Wieland, 1983). Of the three subclasses, only amatoxins are readily absorbed through the digestive tract of humans (Diaz, 2005; Li & Oberlies, 2005); thus, amatoxins (e.g., α‐amanitin) are primarily responsible for fatalities attributed to cyclopeptides (Lindell, Weinberg, Morris, Roeder, & Rutter, 1970). Within *A. phalloides,* 14 amatoxins and phallotoxins are known to occur (Table 1). Both the tissue type and life stage of the mushroom influence the concentration of these toxins. For example, Enjalbert, Gallion, Jehl, and Monteil (1993) determined that α‐amanitin constitutes 17% of the toxin load in the cap, but only 8.8% of the toxin load in the bulb (base of the stem).

**Table 1:** Cyclopeptide toxins previously identified in *Amanita phalloides* mushrooms. Includes the toxin subclass, molecular weight, and source that reported the occurrence

Subclass	Toxin	Molecular weight (Da)
Amatoxin	α‐amanitin^a^, ^b^	918.97
	β‐amanitin^a^, ^b^	919.954
	γ‐amanitin^b^	902.970
	δ‐amanitin^b^	Structure Unknown
	ε‐amanitin^b^	903.962
	Amanin^b^	903.962
	Amanullin^b^	886.979
Phallotoxin	Phalloidin^a^, ^b^	788.868
	Phalloin^b^	772.868
	Phallisin^b^	804.867
	Prophalloin^c^	756.869
	Phallacidin^a^, ^b^	846.904
	Phallacin^d^, ^e^	830.904
	Phallisacin^a^, ^d^	862.903

^a^ Toxins found in highest concentrations in *A. phalloides* (Enjalbert et al., 1992).

^b^ Wieland (1968a).

^c^ Munekata, Faulstich, and Wieland (1978).

^d^ Faulstich, Brodner, Walch, and Wieland (1975).

^e^ Wieland (1983).

While *A. phalloides* and other mushrooms that contain cyclopeptides are lethal to many eukaryotic organisms, at least 17 mushroom‐feeding *Drosophila* species (flies) use these toxic mushrooms and other nontoxic species as developmental hosts (Jaenike & James, 1991; Lacy, 1984). Very little is known about cyclopeptide tolerance in these fly species (reviewed in Scott Chialvo & Werner, 2018). Furthermore, studies examining this adaptation focused exclusively on the effect of α‐amanitin (Jaenike, Grimaldi, Sluder, & Greenleaf, 1983; Spicer & Jaenike, 1996; Stump, Jablonski, Bouton, & Wilder, 2011). Given that *A. phalloides* and other toxic hosts contain a complex mixture of cyclopeptide toxins, we are interested in characterizing toxin tolerance by feeding the flies a diet that contains the full suite of toxins. However, an exhaustive and efficient extraction protocol is needed in order to complete these ecological experiments.

Previous authors (Enjalbert, Gallion, Jehl, Monteil, & Faulstich, 1992; Hallen, Watling, & Adams, 2003) had developed an extraction protocol for toxic *Amanita* mushrooms. However, this “Classic” protocol (described in detail in the Section 2.2.2) was only optimized to extract polar cyclopeptides, disregarding less polar metabolites including some phallotoxins present in the sample. Based on our findings, we report: (a) the development of an exhaustive extraction protocol for cyclopeptides and other secondary metabolites in *A. phalloides*, and (b) propose recommendations for best practices when utilizing host metabolite extracts in ecological experiments based on our work with *A. phalloides*.

## 2 MATERIALS AND METHODS

### 2.1 Mushroom collection, drying, and storage conditions

We collected the *A. phalloides* used in this study at Bolinas Ridge in Nicasio, California (GPS: 38.042664, −122.784958) on December 17, 2017. The identity of the mushrooms was confirmed by Trent Pearce (Naturalist at East Bay Regional Park) and Paul Ginsberg (University of Georgia). The mushrooms were sliced into pieces approximately 6mm thick, and then dried overnight in a Fisherbrand Isotemp General Purpose Heating and Drying Oven at 60°C. We stored the dried mushrooms in a sealed plastic bag at 4°C. Vouchers of these samples are stored at 4°C in Dr. Clare Scott Chialvo's lab (Appalachian State University).

### 2.2 Extraction protocols

For both the “Classic” and Accelerated Solvent Extractor (Thermo Fisher) protocol, the mushroom tissue was prepared in an identical fashion. Approximately 100 mg of dried mushroom tissue with the addition of 1 µg of microcystin‐LR (Internal standard; obtained from Cayman Chemical) was frozen with liquid nitrogen and subsequently macerated using a mortar and pestle. Microcystin‐LR, a cyclic heptapeptide toxin produced by the blue‐green alga *Microcystis aeruginosa* (Eriksson et al., 1989), was added to all the extracted samples to confirm exhaustive extraction of cyclopeptides, which are chemically similar to this internal standard. The ground tissue was then prepared for extraction with either the accelerated solvent extraction (ASE) or “Classic” approach outlined below. For each analysis, three biological replicates of 100 mg of dried mushroom tissue were prepared independently and analyzed.

#### 2.2.1 Accelerated solvent extraction approach

Accelerated solvent extraction (ASE) is an innovative technique using optimized and controlled temperature and pressure conditions to enhance the extraction of numerous solid and semisolid matrices (Richter, Jones, Ezzell, & Porter, 1996). The enhanced yields are obtained by completing the extraction at temperatures exceeding the solvent's boiling point by utilizing increased pressure. Furthermore, the extraction is a fully controlled process, which eliminates the problem of batch to batch variability caused by changing extraction conditions. Thus, ASE is a fast, efficient, and more environmentally friendly approach and has been proven to be a reliable and reproducible extraction technique for numerous types of natural matrices (Borges et al., 2019; Ligor, Ratiu, Kielbasa, Al‐Suod, & Buszewski, 2018; Widelski et al., 2018).

In our ASE approach, the ground mushroom tissue (~100 mg) was mixed with sand to allow proper dispersion of the substrate material. Subsequently, the material was loaded into the 34 ml extraction cell and the sample was extracted first with methanol:water mixture (5:4, v/v) followed by pure methanol. The optimal extraction parameters were determined by testing solvent type (water, methanol, ethyl acetate, n‐hexane), oven temperature (range between 90 and 140°C), number of static cycles (from 1 to 3 cycles), and static time (5 min; 10 min) to acquire the maximum yield in minimal time. Four solvents differing in polarity were used to extract compounds with various physicochemical properties. When using ASE, the polarity of solvent can vary depending on the temperature and pressure used. For example, water, a polar solvent has been shown to be also suitable to extract less polar components from solid matrices when using ASE (Sun, Ge, Lv, & Wang, 2012). All the extracts obtained with different solvents were monitored using HPLC‐DAD‐MS, which allowed to determine the optimal conditions required to extract compounds with varying physicochemical properties.

#### 2.2.2 “Classic” approach

The “Classic” approach follows the methodology laid out by Enjalbert et al. (1992) and Hallen et al. (2003). The ground tissue (~100 mg) was placed in a 15 ml Falcon Tube (VWR), and 5 ml of the extraction solvent (methanol:water:0.01 M HCl 5:4:1 v/v) was added. The tube was incubated overnight at room temperature on a shaker plate. Following the incubation period, the tube was centrifuged for 5 min at 10,000 *g*. The supernatant was decanted into a round‐bottom flask and dried down in a rotary evaporator. It was then resuspended in 2 ml 1:1 methanol:water, transferred to a 2 ml microcentrifuge tube and dried down in a vacuum centrifuge. The resulting solids were resuspended in 1 ml of Millipore water. The reconstituted sample was characterized using HPLC‐MS (protocol detailed below). We also assessed the impact of repeating this procedure on the same tissue pellet by resuspending the pellet in a second volume of extraction solvent (15 ml) after the initial extraction. The pellet was again incubated at room temperature on a shaker plate, and the extraction protocol was repeated exactly 24 hr later.

### 2.3 HPLC‐MS conditions

The *A. phalloides* extracts were analyzed using Agilent 1260 Infinity II system, comprised of an Infinity II Binary Pump, Infinity Multisampler, Multicolumn Thermostat, Diode Array Detector (DAD, UV spectrometer), Agilent's Instrument Control Framework, and Mass Selective Detector (MSD, mass spectrometer). The analysis was performed using Pursuit 5C18 150 × 4.6 mm column at 23°C. The mobile phase comprised (A) water containing 0.2% formic acid, and (B) acetonitrile containing 0.2% formic acid. The following elution gradient was applied: 0–4 min 10% B; 4–20 min 10%–55% B; 20–25 min 55% B; 25–30 min 55%–100% B; 30–33 min 100% B; 33–35 min 100%–10% B; 35–40 min 10% B. Five microlitre of the extract was injected onto the column and run at a 1.0 ml/min mobile phase flowrate. The eluate was monitored by DAD at 214 and 295 nm, and UV‐VIS spectra within the range of 190–400 nm were stored for all the peaks. All the samples were analyzed on the MSD in electrospray positive ionization mode with 10 L/min drying gas flow rate, 50 psig nebulizer pressure, 350°C drying gas temperature, and 4,000 V capillary voltage. For fingerprinting, full scan acquisition mode was used with scan range: 100–1,200.

### 2.4 Identification of cyclopeptide markers

Standard samples of α‐amanitin, β‐amanitin, phalloidin, and phallacidin obtained from SantaCruz Biotechnology (α‐amanitin) and Cayman Chemical (all others). These four cyclopeptides are the only ones with commercially available standards to the best of the author's knowledge. They were dissolved in double‐distilled water (α‐amanitin, β‐amanitin) or methanol (phalloidin, phallacidin) at a concentration of 1 µg/µl. These stock solutions were subsequently diluted ten‐fold and used to confirm identification of selected cyclopeptides in the analyzed extracts.

## 3 RESULTS

### 3.1 Accelerated solvent extraction optimization

**Figure 1:**
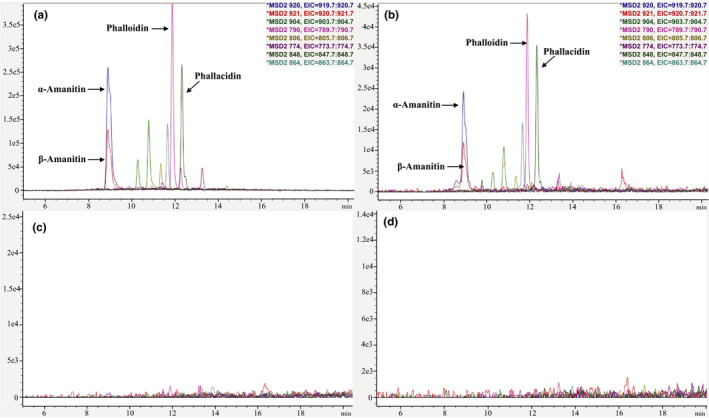
Chromatograms of *Amanita phalloides* samples with the extracted ions of selected cyclopeptides obtained after each consecutive accelerated solvent extraction step using the following solvents or solvent mixtures in sequence: (a) methanol:water mixture (5:4, v/v); (b) methanol; (c) methanol:ethyl acetate mixture (5:4, v/v); (d) ethyl acetate. The amount of material extracted decreases with each extraction step. The axis scale is set by LC/MS ChemStation Software (Agilent) to optimize visibility

We first used an innovative sample preparation technique, accelerated solvent extraction (ASE), to extract secondary metabolites from the *A. phalloides* mushroom samples. The following ASE parameters were found as optimal and used during both accelerated solvent extraction steps: oven temperature—120°C; static time—5 min; static cycles—1; rinse volume—50%. We found that running two consecutive optimized cycles per sample with increasingly less polar solvents (i.e., 5:4 methanol:water followed by pure methanol) resulted in exhaustive extraction of polar toxins as well as less polar metabolites (Figure 1a,b). Additional consecutive rounds of extraction with increasingly less polar solvents (5:4 methanol:ethyl acetate and ethyl acetate) did not recover additional metabolites (Figure 1c,d).

### 3.2 Comparison between “Classic” and ASE protocol

**Figure 2:**
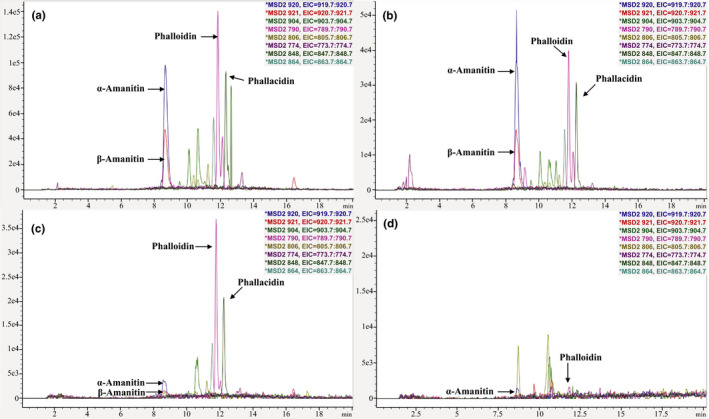
Chromatograms of *Amanita phalloides* samples with the extracted ions of selected cyclopeptides: (a) classical procedure, metabolites obtained from the first extraction; (b) classical procedure, metabolites obtained from completing a second, consecutive extraction on mushroom sample; (c) extract obtained by accelerated solvent extraction of the tissue pellet after one round of the classical procedure; (d) extract obtained by accelerated solvent extraction of the tissue pellet after two classical rounds. The axis scale is set by LC/MS ChemStation software (Agilent) to optimize visibility

We compared the “Classic” procedure to our ASE protocol (Figure 2). First, the dried supernatant generated from the “Classic” approach following the 12‐hr incubation period with methanol:water:0.01 M HCl 5:4:1 v/v was resuspended in water and analyzed on the Agilent 1260 Infinity II system (Figure 2a). We then conducted a second extraction on the remaining mushroom tissue using our ASE protocol (Figure 2c). We found that the “Classic” method failed to exhaustively extract the cyclopeptides in the mushroom sample, with measurable amounts of the less polar cyclopeptides remaining in the mushroom pellet.

Some applications using the “Classic” extraction protocol repeat the extraction procedure twice on a single sample. Therefore, we completed two consecutive rounds of the “Classic” extraction method on a mushroom sample and then extracted the remaining mushroom tissue a third time using the ASE approach. We found that the extract from the second cycle of the “Classic” approach also contained substantial amounts of cyclopeptides (Figure 2b), and measurable quantities of the cyclopeptides were still recovered by ASE extraction (Figure 2d).

**Figure 3:**
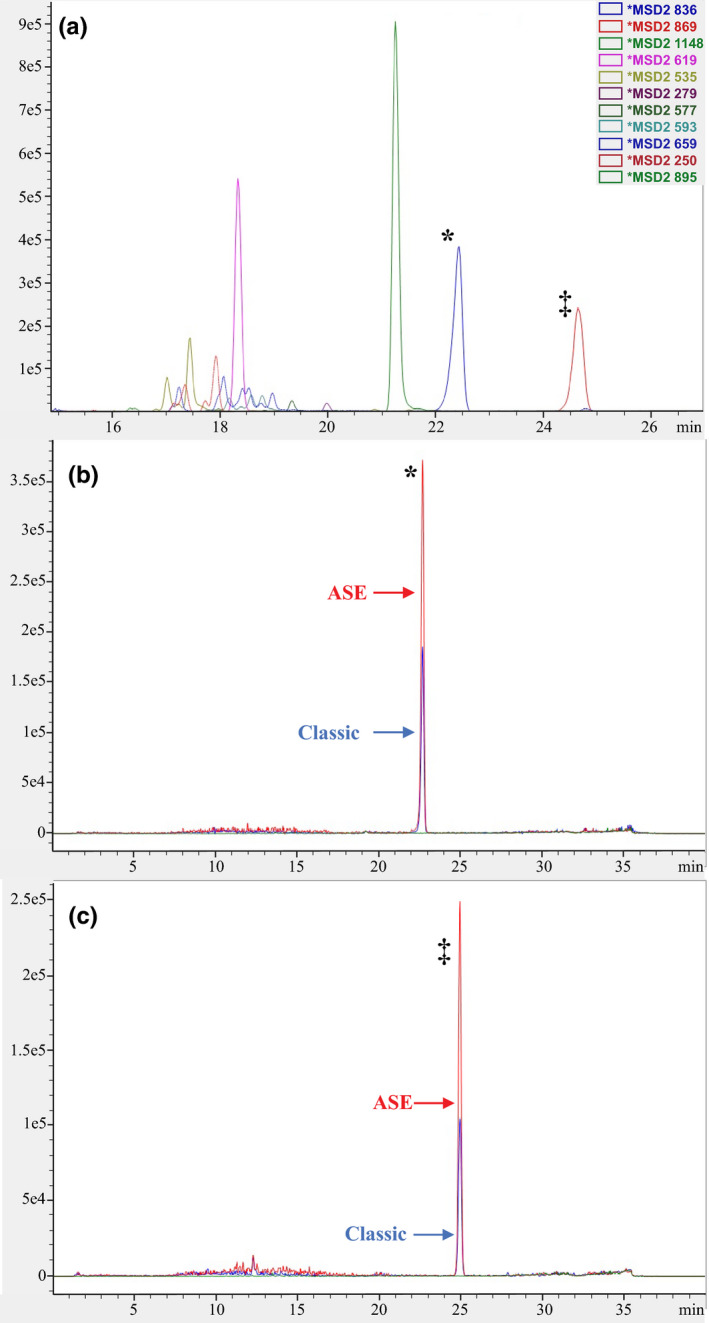
Comparison of extraction efficiency between “classic” and ASE techniques for less polar compounds (a) peaks of selected less polar cyclopeptides extracted with methanol in the second step of accelerated solvent extraction; (b) comparison of the two techniques for less polar cyclopeptides eluting at ~22.3 min; (c) comparison of the two techniques for less polar cyclopeptides eluting at ~24.6 min

The solvents used in the “Classic” method (i.e., methanol:water:0.01 M HCl 5:4:1 v/v) are optimized for the isolation of more polar cyclopeptides (e.g., α‐amanitin). In addition to exhaustively extracting the polar toxins, we found that our ASE extraction protocol isolated almost double the amount of less polar compounds than the “Classic” procedure (Figure 3).

## 4 DISCUSSION

Previously, several papers described extraction protocols for α‐amanitin and other cyclopeptides (Enjalbert et al., 1992; Hallen et al., 2003). To the best of the authors' knowledge, all published procedures focus on extraction of only the polar cyclopeptides by incubating macerated mushroom tissue for 12–24 hr in aqueous‐methanolic solutions with the addition of mineral or organic acid (e.g., hydrochloric or formic acid). Some protocols (Deng et al., 2011; Hallen, Adams, & Eicker, 2002) call for re‐extraction of the remaining pellet, extending the procedure to 48 hr. We compared this procedure to the ASE protocol and demonstrated that this “Classic” approach was not exhaustive even following double extraction of the mushroom samples. Furthermore, the extract resulting from the “Classic” protocol was composed primarily of the polar fraction of metabolites and misrepresents more lipophilic metabolites. These less polar compounds, such as antamanide, a possible antitoxin for some *Amanita* cyclopeptides (α‐amanitin and phalloidin; Wieland, 1968b), could alter the observed biological effect of the complete suite of secondary metabolites relative to a single metabolite of interest (e.g., α‐amanitin). Thus, these data indicate that previous reports may have underestimated the amount of amanitin and other toxins in mushroom tissue.

The results of our work add to a growing body of literature demonstrating the use of ASE for the extraction of many classes of secondary metabolites (Borges et al., 2019; Ligor et al., 2018; Widelski et al., 2018). To the best of our knowledge, this is the first report on exhaustive extraction of cyclopeptides and other metabolites from *Amanita* samples. The optimized ASE procedure significantly shortened the process of sample preparation preceding feeding assays. The use of ASE provided extracts more closely resembling the chemical complexity of real samples. ASE can be easily optimized for any type of solid matrices and improve batch to batch reproducibility. Thus, ASE could be used in a broad range of ecological experiments examining different plant‐insect interactions.

## 5 EXPERIMENTAL BEST PRACTICES

**Figure 4:**
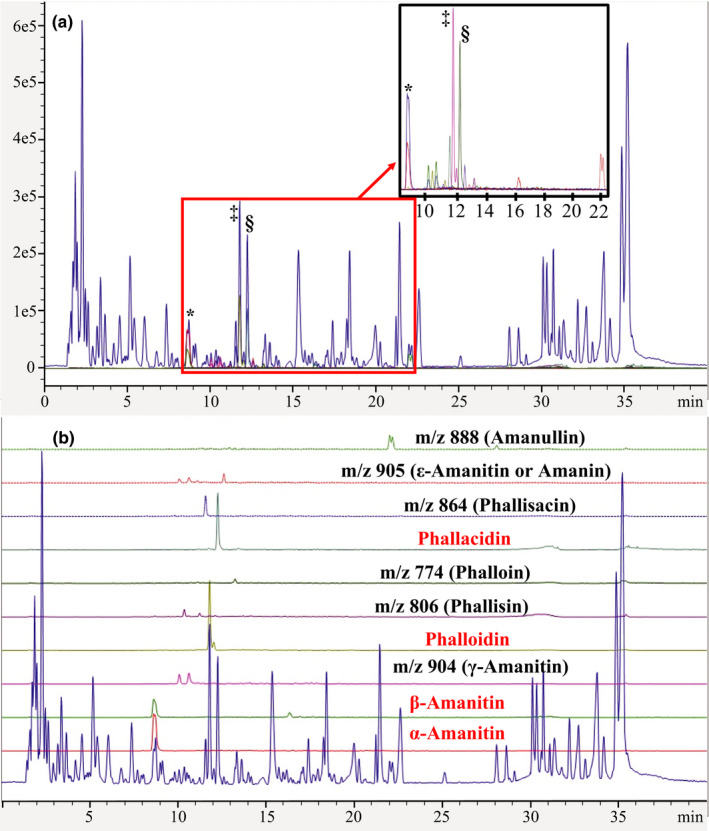
Chromatographic fingerprint of *Amanita phalloides* sample extracted using accelerated solvent extraction protocol. (a) Peaks of commercially available standards: *α‐amanitin + β‐amanitin; ^‡^phalloidin; ^§^phallacidin; (b) peaks for 10 compounds we surveyed for in the toxin extract

In order to maximize the reproducibility of our work and that of other researchers using host secondary metabolite extracts in experiments examining plant‐insect interactions, we make the following recommendations for best practices. (a) When a host secondary metabolite extract is used within an experiment, be sure to include specific details of the precise manner in which the extract was produced. Important points to identify include where, when, and how tissue samples were originally gathered, whether the natural products were extracted from fresh, frozen, or dried material, the method used to prepare the sample for extraction, the extraction protocol and its parameters, and the method of storage following extraction. (b) Following the production of the secondary metabolite extract, it should be fingerprinted prior to use. We recommend fingerprinting the sample using markers of compounds known to occur in the extract, which allows for the identification and quantification of the amounts of different metabolites. In this study, we used four commercially available standards for cyclopeptides (Figure 4). The full fingerprint of the analyzed extract demonstrates the complexity of the sample, a factor that has not been addressed in previous studies of toxin tolerance (Jaenike et al., 1983; Spicer & Jaenike, 1996; Stump et al., 2011). Furthermore, having a fingerprint of previous sample extracts makes it possible to compare different batches. These comparisons can then be used to determine whether the extracts differ significantly. (c) To maximize consistency, the manner in which the secondary metabolite extract will be used in an experiment should be clearly specified. By using the extract as opposed to fresh host tissue, it is possible to control the quantities of the different metabolites to which the insects are exposed.

## 6 CONCLUSIONS

With the recent drive for studies examining plant‐insect interactions to consider the impact of complex mixtures of host secondary metabolites on insects (Dyer et al., 2018), there is a need for exhaustive extraction protocols and detailed methodologies that allow for experimental replication. The results of our study highlight the importance of assessing the efficacy of previously designed protocols. In particular, protocols should be assessed for their ability to recover all secondary metabolites from a sample. For the extraction of cyclopeptide toxins from *Amanita* mushrooms, our extraction protocol is both comprehensive and also substantially faster than previously used methods. We make the following three recommendations for best practices when working with host secondary metabolites in experiments: (a) specify the extraction protocol for the secondary metabolite(s) being used in an ecological experiment, (b) fingerprint the extract prior to use in feeding assays to account for differences between different extracts, and (c) describe in detail the exact manner in which the host extract is used in the experiment.

## CONFLICT OF INTEREST

None declared.

## AUTHOR CONTRIBUTIONS

**Clare H. Scott Chialvo**: Conceptualization (equal); funding acquisition (equal); investigation (equal); resources (equal); supervision (equal); visualization (equal); writing‐original draft (equal); writing‐review and editing (equal). **Logan H. Griffin**: Data curation (supporting); investigation (supporting), writing‐review and editing (supporting). **Laura K. Reed**: Conceptualization (equal); funding acquisition (equal); resources (equal); validation (equal); writing‐review and editing (equal). **Lukasz Ciesla**: Conceptualization (equal); data curation (lead); formal analysis (lead); investigation (equal); methodology (lead); resources (equal); software (lead); supervision (equal); validation (equal); visualization (equal); writing‐original draft (equal); writing‐review and editing (equal).

## Data Availability

The HPLC spectral data are deposited in Dryad under https://doi.org/10.5061/dryad.47d7wm398.
